# Contexts and Parental Management Strategies for Child-to-Parent Violence in Adolescents with Attention-Deficit/Hyperactivity Disorder: A Qualitative Study

**DOI:** 10.3390/children12040483

**Published:** 2025-04-09

**Authors:** Chia-Fen Wu, Ching-Shu Tsai, Yu-Ping Chang, Fan-Hao Chou, Cheng-Fang Yen

**Affiliations:** 1 School of Nursing, Kaohsiung Medical University, Kaohsiung 80743, Taiwan; u111840001@kmu.edu.tw; 2 School of Medicine, College of Medicine, I-Shou University, Kaohsiung 82445, Taiwan; jingshu@cgmh.org.tw; 3 Department of Child and Adolescent Psychiatry, Chang Gung Memorial Hospital, Kaohsiung Medical Center, Kaohsiung 83341, Taiwan; 4 School of Nursing, The State University of New York, University at Buffalo, New York, NY 14214-8013, USA; yc73@buffalo.edu; 5 Department of Psychiatry, Kaohsiung Medical University Hospital, Kaohsiung 80708, Taiwan; 6 Department of Psychiatry, School of Medicine, College of Medicine, Kaohsiung Medical University, Kaohsiung 80743, Taiwan

**Keywords:** adolescent, attention-deficit/hyperactivity disorder, child-to-parent violence, parent

## Abstract

Background/Objectives: This qualitative study was conducted to understand the experiences of parents of adolescents with attention-deficit/hyperactivity disorder (ADHD) regarding experiences of child-to-parent violence (CPV), including the contexts of parent–child conflict in which CPV occurred, types of CPV, victimized parents’ feelings and responses to CPV, and parents’ strategies for better handling of CPV based on past experiences. Methods: Data from open and in-depth interviews with 18 parents who have experienced CPV perpetrated by their children with ADHD were subject to reflexive thematic analysis. Results: The results revealed various contexts in which parent–child conflicts (CPV) occurred. Parents reported the experiences of psychological aggression, physical aggression, and restrictions on movement. In addition to experiencing feelings of distress, fear, and frustration, parents adopted various strategies for coping with adolescents’ CPV, such as leaving the scene, pushing back, rational communication, controlling their own emotions, encouraging their children to express their feelings, and seeking help. Parents suggested that practicing communicating with their children using real-life examples and learning parenting skills is essential to the prevention of CPV and the mitigation of serious consequences. Conclusions: Our findings can provide healthcare professionals with key insights into the contexts of CPV and the management strategies used by parents of adolescents with ADHD.

## 1. Introduction

Child-to-parent violence (CPV) refers to acts of verbal, physical, emotional, or financial control carried out by children against their parents [1]. Studies on CPV have rapidly grown in recent years [2], indicating the importance of CPV to the health of children and parents [3]. A meta-analysis demonstrated that one-fourth of children in Latin America have perpetrated psychological violence and 5% to 6% have perpetrated physical violence against their parents [4]. Another review found that the proportion of physical CPV was 5% to 21% among children in the community [5]. CPV can affect children and their parents in several ways [3]. First, CPV can threaten the safety of parents and other family members. Victimized parents’ self-defense behaviors or countermeasures may also result in injury to the children perpetrating CPV. Second, victimized parents may feel shame and helplessness as a result of being insulted or controlled by their child. The shame associated with CPV may cause delays in parents seeking help [6,7,8]. Third, parents may feel that they have failed to discipline their child and are no longer able to practice effective parenting. Moreover, CPV may not be addressed in time and may lead to the development of more serious health-threatening behaviors or crimes being committed. Therefore, such behaviors need to be investigated and prevented.

Studies have reported a high proportion of adolescents with attention-deficit/hyperactivity disorder (ADHD) perpetrating CPV [9,10,11], with several potential reasons for this. First, adolescents with ADHD have differences in cognitive functioning [12], social functioning [13], and information processing [14] compared to other adolescents. These neurodevelopmental dysfunctions may be detrimental to impulse control in adolescents who are involved in disputes with their parents. For example, a study in the United States found that executive function impairment was uniquely associated with physical aggression in children with ADHD [15]. For boys with ADHD, poor executive function was also indirectly associated with greater physical aggression through the expression of ADHD behaviors [15]. A deletion/insertion polymorphism within the 5-HT transporter (5-HTT) promoter gene increases the risk of physical violence in men with a history of ADHD [16]. In addition, a high proportion of children with ADHD have comorbid oppositional defiant disorder (ODD), characterized by disruptive behavior, patterns of anger and irritable mood, being argumentative, and vindictive behavior [17]. Children with ADHD also have a higher risk of comorbid conduct disorder (CD), characterized by a consistent pattern of aggressive and disobedient behaviors [17]. Both comorbid ODD and CD worsen ADHD symptom severity and are associated with high psychosocial dysfunction, including verbal or physical aggression toward family members [18]. Studies have demonstrated that there are contexts and forms of conflict between adolescents with ADHD and their parents that differ from those of other families [9,19]. For example, adolescents with ADHD engage in more risk-taking behaviors, such as cell phone and internet overuse, than other adolescents, which may trigger parental concern and control [20,21]. Moreover, compared to adolescents without ADHD, a higher proportion of adolescents with ADHD are exposed to violence at an early age, which increases the risk of attacking others in the future [22]. A scoping review found that children and adolescents with disabilities of any kind have been excluded from most studies on CPV and that there are very few qualitative studies on this type of violence [2]. Therefore, it is of clinical importance to conduct qualitative studies to understand the experiences of parents of adolescents with ADHD when faced with CPV.

Scenarios in which CPV occurs involving adolescents with ADHD and parental management strategies to address it are particularly important areas to explore in qualitative studies. CPV arises from conflicts between children and their parents. According to conflict management strategy theories [23,24,25], parent–child conflict can occur when there is disagreement about the value, process, or evaluation of tasks such as completing chores or rules around cell phone use. Each conflict situation calls for a different conflict management approach [23]. Understanding the most common scenarios that lead to CPV is the first step to conceptualizing this type of violence and helps parents handle CPV [26]. Further, parents may have various responses to and strategies to manage CPV, leading to different results [27,28,29,30]. For example, problem solving is often the best strategy for conflict resolution because it results in an integrative solution that satisfies both parents and children. However, most situations in which CPV occurs do not allow for the application of problem solving to handle disputes. Understanding parents’ experiences of responses to and management of CPV will help to identify appropriate resolution strategies.

This qualitative study explored the experiences of parents of adolescents with ADHD regarding CPV, including the contexts of parent–child conflict in which violence occurs, types of CPV, victimized parents’ feelings and responses to CPV, and parents’ strategies for the effective handling of CPV based on past experiences. We hypothesize that parents of adolescents with ADHD experience various types of CPV in multiple contexts. We also hypothesize that parents have their own feelings about and ways of coping with CPV. This study is valuable given the scarcity of studies exploring this topic in parents of adolescents with ADHD. The findings of the present study can provide valuable insights into parents’ experiences of CPV and management strategies, subsequently informing the development of targeted interventions aimed at reducing CPV in adolescents with ADHD.

## 2. Methods

### 2.1. Participants and Procedure

This qualitative study was conducted from August to December 2024. The parents of adolescents with ADHD who had participated in the Study on Child-to-Parent Violence in Adolescents with ADHD (CPV-A-ADHD), conducted from August 2023 to July 2024, were invited to take part. In the CPV-A-ADHD, parents of adolescents with ADHD were recruited from six child psychiatry outpatient clinics at two hospitals in Taiwan. Parents of adolescents who were 11–18 years of age and had ADHD diagnosed by a certified child psychiatrist in accordance with the Diagnostic and Statistical Manual of Mental Disorders, Fifth Edition (DSM-5) [17] were invited to take part in the CPV-A-ADHD. Parents of adolescents who had comorbid intellectual disability, severe autism spectrum disorder, bipolar disorder, or schizophrenia, according to the DSM-5, were excluded. Parents who had any other cognitive deficits that may impede their understanding of this study’s purposes and completion of the research questionnaire were also excluded. In total, 247 parents of adolescents with ADHD participated in the CPV-A-ADHD. Parents’ experiences of CPV, such as psychological aggression, physical aggression, financial demand, and control or domination in the previous year, were assessed using the CVP-Q, Parents’ Version (CPV-Q-P) [31]. The total score for the CVP-Q-P ranged from 0 to 56. A higher total score indicates a higher frequency of CPV.

From the CPV-A-ADHD, we invited 37 parents who had total CPV-Q-P scores ≥ 10 (ranking in the top 15% of all parents) to participate in this study. In total, 18 parents agreed and participated in this qualitative study. All parents received explanations regarding the objectives and methods of this study and provided written informed consent. The sample size was sufficient for saturation, and no additional themes were generated after completing interviews with 18 parents [32]. Of the participants, 16 were mothers and 2 were fathers; 12 had a bachelor’s degree and 6 had a high school diploma; and the mean age was 45.4 years (standard deviation = 6.2 years).

### 2.2. Ethical Considerations

The protocol of the present study was approved by the Institutional Review Boards of Kaohsiung Medical University Hospital (KMUHIRB-SV(II)-20210113) and Chang Gung Memorial Hospital, Kaohsiung Medical Center (202102157A3C601). To safeguard participant privacy and uphold human rights, the participants were provided with detailed information regarding the study objectives, privacy and security measures, and their right to withdraw from this study at any time. The interviews were audio recorded only after participant consent had been obtained. All collected data were coded anonymously and stored securely to ensure complete confidentiality.

### 2.3. Data Collection and Analysis

Open-ended interviews were conducted to gather comprehensive data regarding both objective and subjective experiences from the participants. Each interview began with a central question, followed by the introduction of clarifying subquestions and follow-up prompts as needed [32]. Each interview comprised the following questions: (1) In the previous survey, you mentioned that you had been physically or verbally attacked by your child in the past year. Can you recall what happened at that time? What kind of aggressive behavior did your child exhibit to you? (2) Were you and your child discussing anything before your child attacked you? (3) How did you react? What was the outcome? (4) Would you have handled the situation differently if you had done it all over again?

To establish circumstantial intersubjectivity during each interview, the interviewer scheduled a convenient time and reserved a suitable research room for the participant. Before starting the interview, the interviewer provided the participant with an informed consent form, which was reviewed in detail to ensure that the participant fully understood and agreed to the terms and criteria of the research. This process ensured transparency regarding this study’s scope, participant rights, and confidentiality procedures to foster an environment of trust and mutual understanding. Additionally, demographic information—including the participants’ age and educational level—was collected during this initial phase.

The interviews were conducted by an experienced researcher enrolled in a doctoral program. Each interview lasted 45–60 min. All of the interviews were audio recorded and subsequently transcribed verbatim to ensure accuracy and completeness. To further enhance the reliability of the data, the transcriptions were cross-checked for accuracy by a second researcher; this additional verification step helped us to identify and correct any potential errors or omissions in the transcription process and thus ensure that all of the data accurately reflected the participants’ responses.

The six-phase reflexive thematic analysis framework developed by Braun and Clarke [33,34] was employed to analyze the interview data. The six phases are described as follows: (1) Dataset familiarization and immersion: The researchers thoroughly reviewed and rereviewed the interview transcripts to gain a deep understanding of the participants’ experiences. (2) Identifying and organizing segments of data with similar meanings or meaning patterns into codes: This step included the systematic coding of notable features of the data across the entire dataset. (3) Generating initial themes that shared a core idea or concept: In this step, codes were grouped into potential themes on the basis of shared core ideas. All relevant data segments were then collated under each potential theme. (4) Developing and reviewing themes: This step involved refining the initial themes by revisiting the transcripts to ensure that the initially identified themes accurately captured the participants’ narratives and reflected the essence of the data. (5) Refining, defining, naming, and finalizing themes: In this phase, themes were further refined, defined, and named to ensure their clarity and distinctiveness. This step ensured that the final themes accurately represented the data. (6) Report writing and thematic map development: The research findings were presented as a comprehensive report. The final thematic map ensured that the reported themes remained true to the data and effectively conveyed the story revealed by the participants’ experiences.

Two researchers independently reviewed the transcripts line by line and paragraph by paragraph to identify notable statements and coded them on the basis of recurring patterns and concepts related to the interview questions. The researchers wrote down their initial thoughts on memos and then summarized the memos as codes. After a review of the first two participants’ transcripts, the two researchers reviewed another reviewer’s coding results and held a meeting. Through discussion, the researchers gave feedback to each other regarding how to modify the coding and thereby enhance the interrater reliability. After reviewing all transcripts, the researchers then compared codes, resolved discrepancies, and grouped the codes into potential themes on the basis of shared core ideas. The researchers also used each other’s analytic memos as a reference for revising the themes. These themes were then further combined or revised into overarching themes, with several subthemes supporting each main theme. This iterative process was continued until the two researchers reached a 100% consensus regarding the meaning of the coded data for the entire dataset and the adequate representation of themes. Throughout this process, the researchers wrote memos documenting reflections, thoughts, and insights related to the data and the coding process. This process ensured a thorough and transparent analysis and enhanced the rigor of this study through reflexivity.

## 3. Results

Four main themes and 24 subthemes were obtained from the interview data (Table 1).

### 3.1. Theme 1: Contexts of Parent–Child Conflicts in Which CPV Occurred

The participants reported various contexts of parent–child conflict in which CPV occurred.

#### 3.1.1. Resistance or Procrastination in Completing Tasks

When parents ask their teenage children to carry out chores, conflicts may arise if the children are unwilling to comply, sometimes even escalating into violent behavior toward the parents. Participant 3 said

*“The most common scenario is when you ask him to do something he doesn’t like—like cleaning his room—he would start yelling at me.”*

Participant 8 said

*“Whenever I tell him to tidy up his room or remind him to complete his daily tasks, he would get upset. His temper would suddenly flare up, and he would start throwing things—whatever he could grab nearby—at me.”*

Teenagers often procrastinate when reminded of their responsibilities. If parents repeat their reminders, it can trigger verbal aggression from the child. Participant 14 said

*“He always procrastinates, so I have to remind him repeatedly. He would say, ‘Just a moment, just a moment’. And if it’s something he really doesn’t want to do—like chores or reviewing schoolwork—he would drag it out even longer. Every time I remind him again, he would lose his temper and start hurling insults.”*

#### 3.1.2. Fighting Against Cell Phone Use Control or Daily Routines

Parents’ control over teenagers’ cell phone usage is one of the most frequently mentioned contexts of parent–child conflicts, often leading to violent reactions from the child. Participant 5 said

*“I allow him to use his cell phone but set a time limit. He often argues with me when he goes over the time limit. I would tell him, ‘Alright, time’s up, hand over the phone’, and he would ask for more time, saying, ‘Just let me finish this round’. But what normally happens is that he would lie to me that he hasn’t finished the round even though he has. One time, I caught lying, and I got really angry. I took the phone away from him, and he completely lost it, yelling at me furiously.”*

Participant 13 said

*“Lately, he’s been obsessed with his cell phone. If he asks for it to play games and I say no, he would provoke me deliberately, throw a tantrum, and storm back into his room, slamming the door shut.”*

Participant 4 said

*“When I first bought him a cell phone, I set restrictions on usage time. But he constantly argues with me over it—sometimes even resorting to physical force to snatch the phone from me.”*

Beyond cell phone usage, conflicts also arise when parents ask children to stop their hobbies and rest instead. Participant 3 said

*“Last year, he was obsessed with magic tricks. He would stay up past midnight practicing. One night, I heard noises coming from his room, so I went in and told him to turn off the lights and go to bed. He got angry.”*

#### 3.1.3. Perceived Parental Favoritism Toward Siblings

Parents may apply different rules and act differently based on their children’s ages. However, teenagers may perceive these differences as being unfair, leading to parent–child conflicts. Participant 1 said

*“He was the only child before, so he considered his room and toys solely his, and no one else was allowed to touch them. When his younger brother grew older and started using his things, he would often lose his temper.”*

Participant 5 said

*“He frequently changes the rules and makes his younger sister comply with them, and he has a strong desire to win. For example, he would invite her to play a game, but if she wins, he would get upset and change the rules, forcing her to replay. Sometimes, if she’s watching TV, he would snatch the remote and say, ‘Why is it always you watching?’ When I step in to correct him, he would accuse me of favoring his sister.”*

Participant 10 said

*“She thinks we’re biased—that we only discipline her and not her younger sisters. She feels it’s unfair when we ask her to do things but not her sisters. This often leads to her breaking down in tears and throwing a tantrum.” (case 010)*

#### 3.1.4. Unmet Material Desires

Teenagers often desire certain things but lack the financial means to purchase them themselves. When their parents refuse to buy them for them, conflicts may arise. Participant 3 said

*“He is very materialistic—he wants everything he sees. For example, we once went to a stationery store, where he demanded that I buy him pens. I told him he had plenty and refused to buy them for him. When we got home, he started yelling at me.”*

Participant 6 said

*“He would constantly ask for electronic products such as iPads or an Apple Watches—basically, anything made by Apple. Of course, I would say no because I know he doesn’t really need them; he just wants to follow the trend and show off. However, this would always lead to loud arguments.”*

#### 3.1.5. Unfiltered or Emotionally Charged Discipline

Every parent has their own ways of disciplining their children. However, if parents struggle with emotional control or resort to harsh, emotionally charged language, teenagers may adopt the same behavior in response. Participant 11 said

*“When I help him with his homework, I would sometimes lose my temper and say things like, ‘Why are you so dumb? Is there something wrong with your brain that prevents you from doing this?’ Now, he uses the same words to insult me.”*

Participant 12 said

*“Whenever his dad asks him to do chores, he would either procrastinate or say no. If his dad happens to be in a bad mood or has been drinking, he would snap and yell, ‘Why do you always say “in a minute”? Do it now!’ As our child grew older, conflicts between them became more frequent.”*

### 3.2. Theme 2: Types of CPV

During parent–child conflicts, teenagers may exhibit various forms of aggression toward their parents, ranging from verbal attacks and property destruction to direct physical violence and even restricting their parents’ movement.

#### 3.2.1. Verbal Abuse or Threats

Verbal abuse and threats are the most common forms of aggression that teenagers direct toward their parents. Participant 5 said

*“He often loses his temper with me and says hurtful things like, ‘You’re a terrible mom, it’s all your fault’, ‘Why don’t you just die?’ or ‘I might as well be dead’.”*

Participant 8 said

*“When he gets furious, he would yell at me, hurl profanities, and insult me with demeaning or threatening remarks like, ‘You better not let me see you again’, or ‘Why don’t you just drop dead?’.”*

#### 3.2.2. Property Destruction

Teenagers with poor impulse control may resort to destroying objects as an outlet for their anger. Participant 3 said

*“When I refuse to buy something for him, he would come home and start shouting at me, smashing objects around the house and violently slamming things.”*

Participant 2 said

*“During a few conflicts, he got so angry that he grabbed a stick and pointed it at me—not to hit me, but to stab holes into a cardboard box over and over.”*

Participant 9 said

*“He didn’t want to do the dishes, but instead of saying so, he took a lighter and burned the window screen in his room. I only found out about it when I noticed the burn marks and confronted him.”*

#### 3.2.3. Physical Attacks or Throwing Objects at Parents

Teenagers with poor impulse control may also physically lash out at their parents during heated arguments. Participant 8 said

*“Whenever he gets upset, his temper would explode. He would throw whatever is within reach at me and even slap me.”*

Participant 4 said

*“One time, he was arguing with his mom and suddenly flipped the dining table over. When his mom tried to leave, he ran over and shoved her to the ground.”*

#### 3.2.4. Retaliation Against Harsh Discipline

In response to intense parental discipline or physical punishment, some teenagers may retaliate physically. Participant 3 said

*“He refused to stop doing magic tricks late at night, so I tried to take away his deck of cards. When he wouldn’t let go, I threatened, ‘If you don’t let go, I’ll tear them up so you won’t be able to use them’. He got furious and kicked me off the bed.”*

Participant 12 said

*“He often argues back and forth with his dad. When his dad tries to hit him, he would clench his fists, ready to punch back or push his father.”*

Participant 13 said

*“A few times, I tried to stop him from using his cell phone, but he ignored me. So I picked up a stick and acted like I was going to hit him—not to actually hit him, but just to scare him into stopping. When he saw that, he raised his hand as if he was ready to fight back.”*

#### 3.2.5. Restricting Parents’ Movement

During conflicts, some teenagers may physically prevent their parents from moving in order to get their way. Participant 14 said

*“When he loses his temper, he would retaliate physically. If I try to walk away to avoid an argument, he would grab me or stop me from leaving. Sometimes, he would pull me so hard that I would fall or strain a muscle.”*

Participant 2 said

*“That time, I turned off his internet because the router switch was in my room. He retaliated by blocking me from going downstairs. On one occasion, he even grabbed my hand and forced me to turn the internet back on.”*

### 3.3. Theme 3: Parents’ Feelings and Responses to Their Children’s Violence

When faced with their children’s violent behavior, parents experience/have a range of emotions and reactions. These include feeling emotionally overwhelmed or fearful, avoiding the situations by leaving the scene or adopting a passive coping attitude, responding aggressively in an attempt to stop the violence, engaging in rational communication, empathizing with their children, and helping them manage conflicts with older adults.

#### 3.3.1. Emotional Distress, Fear, and Frustration

Children’s intense emotions and violent behavior can provoke strong emotional reactions in parents, making them react violently either emotionally or behaviorally, or feel frustrated by the challenges of parenting. Participant 6 said

*“I have emotions too, and they are affected by him. When he yells at me, I get even angrier and yell back. I won’t back down—I’ll argue with him or shout even louder.”*

Participant 7 said

*“When we argue, I sometimes lose control and yell back at him. That time, I raised my hand and hit his arm, and he hit me back. We ended up wrestling, where I injured my hand.”*

Participant 14 said

*“During conflicts, I often feel scared and just want to end the arguments quickly. He’s grown taller and stronger now—if it ever turned into a real fight, I wouldn’t be able to defend myself.”*

Participant 12 said

*“His father can’t stand him constantly losing his temper. He doesn’t understand why, after so many doctor visits, he’s still like this… But I can see that he’s making small improvements. I feel stuck in the middle—I don’t know what to do either.”*

#### 3.3.2. Leaving the Scene or Using a Passive Approach

Some parents choose to leave the scene or use a passive approach to prevent conflicts from escalating and to reduce the risk of violence. Participant 6 said

*“When the conflicts get too intense and I can’t take them anymore, I would leave the house to get away from them and calm myself down. Sometimes, I would even turn my cell phone off so that my family wouldn’t be able to reach me.”*

Participant 10 said

*“When my child throws a tantrum, we usually just ignore her. Eventually, she would storm off to her room, lock the door, and continue sulking.”*

#### 3.3.3. Using Strong Reactions to Stop the Child

Some parents try to stop their children’s violent behavior by yelling back or even picking up sticks to assert control. Participant 8 said

*“When he screams at me, I would scream back—not only to vent my own emotions but also in the hope that it would stop the conflict.”*

Participant 13 said

*“When I get really angry, I would take out a stick. I do not actually hit him—I would just hold it and ask, ‘Why are you acting like this?’ to make him stop his outburst.”*

#### 3.3.4. Rational Communication

Some parents are able to maintain rational communication despite their children’s violent behavior. Participant 9 said

*“When he throws things on the floor, I would ask him, ‘What were you trying to express with this action? Tell me’.”*

Participant 11 said

*“Whenever he gets angry because he doesn’t want to do his homework, I don’t react immediately. I take a deep breath to calm myself down, then I say to him, ‘If you don’t do it now, your teacher will still make you finish it tomorrow. The teacher might even call me and ask me watch you do your homework every day. So either way, you’ll still have to do it’.”*

#### 3.3.5. Thinking from the Children’s Perspective

Some parents try to see things from their children’s points of view during conflicts. Participant 1 said

*“He would slam his bedroom door shut and lock it, refusing to let us in. I think this is his way of telling us not to touch his belongings anymore.”*

Participant 9 said

*“When I refuse to buy him something, he acts out or says things to provoke me. But I don’t let it get to me. Instead, I reflect on why he’s behaving this way—what is he trying to achieve?”*

Participant 14 said

*“In the heat of the moment, I know he’s trying to express something, but he might not have the communication skills to make himself clearly understood. So instead, he resorts to actions like grabbing me or blocking my way, as if he’s saying, ‘Don’t go, listen to me’.”*

#### 3.3.6. Helping Children Reduce Conflicts with Older Adults

Some parents try to de-escalate conflicts between their children and other family members. Participant 12 said

*“When I hear my husband and son raising their voices, I would walk over and stand beside my son, trying to steer the conversation in a different direction. If I see that the argument is getting too heated, I would take my son to his room and stay there with him for about half an hour. By then, his anger would usually subside.”*

#### 3.3.7. Seeking Help

When parents find it difficult to handle their children’s violent behavior, they may seek outside assistance. Participant 7 said

*“When he starts throwing things, I would call his father to come home and stop him… I have also called the domestic violence hotline (113) for help before.”*

### 3.4. Theme 4: Strategies for Better Management of CPV Based on Past Experiences

After experiencing their children’s violent behavior, some parents reflect on past incidents and believe that adopting certain strategies in the future may lead to better results.

#### 3.4.1. Controlling Their Own Emotions and Leaving the Scene

Parents believe that managing their own emotions and not reacting with anger are essential when dealing with their emotionally heightened teenage children. One effective strategy is to remove themselves from the conflicts. Participant 8 said

*“If I could redo everything all over again, I would try to control my emotions and handle the situation more calmly to see if the outcome would be different.”*

Participant 4 said

*“If I feel like I’m about to lose my temper, I leave the scene or change my approach to communication instead of confronting him head-on.”*

#### 3.4.2. Avoiding Arguments That Trigger Child Violence

Parents believe that to prevent violent behavior from their teenage children, one essential approach is to avoid conflicts that could provoke aggression, while still maintaining boundaries and not giving in to every demand. Participant 2 said

*“The rules we agreed on for cell phone usage must still be followed. However, I now give him a 10-minute warning before his usage time ends so that he doesn’t explode when I suddenly take his cell phone away.”*

Participant 1 said

*“When mediating arguments between him and his younger brother, I no longer say things like ‘Your brother is still little, you should let him have his way’, because this makes him feel like the adults are being unfair.”*

#### 3.4.3. Analyzing Situations to Help the Child Understand Them

Parents believe that calmly analyzing current situations and helping their teenage children understand them—without adding negative emotions—can help de-escalate conflicts. Participant 3 said

*“I try to lower my voice when explaining issues to my child. I also ask for his perspective instead of turning the issues into confrontations.”*

#### 3.4.4. Encouraging Children to Express Their Feelings

Parents believe that encouraging their teenage children to express their emotions before they reach a breaking point can prevent violent outbursts caused by feeling unheard. Participant 16 said

*“When I notice his mood turning sour and him starting to get worked up, I would encourage him to express his thoughts instead of immediately trying to reason with him.”*

Parents also feel that once emotions have settled after a conflict, having discussions can help both sides understand each other and improve the handling of future conflicts. Participant 18 said

*“After arguments, when we have both calmed down, I would have a talk with him. I would listen to his perspective and explain why I got so upset. This is when reasoning with him actually works.”*

#### 3.4.5. Self-Reflection

Parents believe that self-reflection is crucial—acknowledging whether their own actions have contributed to the conflicts that led to their teenage children acting violently. Participant 7 said

*“Thinking back, I’m already in my 50s, and even I struggle to control my emotions sometimes. How can I expect him to have complete self-control? Maybe I should change myself first and lead by example.”*

Participant 17 said

*“Honestly, I bear a lot of responsibility for his intense emotional reactions. I shouldn’t have confronted him so directly in that situation. The back-and-forth just escalated things further.”*

Participant 11 said

*“Later, he apologized to me, but he also said that I love his older brother more and that I favor him less. I apologized to him too. Regardless of whether we, as parents, actual show favoritism, if he feels like he is treated unfairly, then I believe we should acknowledge that and apologize.”*

#### 3.4.6. Practicing Communication in Everyday Life

Parents believe that parent–child communication should not only start after violent incidents occur. Instead, they should take opportunities in their daily lives to practice handling conflicts. Participant 11 said

*“When we’re not arguing, I use examples from TV shows, movies, or real-life events to discuss with my child: ‘If this happened in our home, how should we handle it?’ Having these discussions in advance acts as practice, helping us manage our emotions better when conflicts occur.”*

#### 3.4.7. Strengthening Parenting Skills

Parents recognize the need to improve their own parenting skills in order to handle conflicts effectively and to prevent violent outbursts from their teenage children. Participant 4 said

*“Because our child is so emotionally intense, his mother and I have read books, attended group courses, and even sought counseling—all to build a better relationship with him and support his growth. We’ve made a lot of changes for his sake.”*

## 4. Discussion

Parents reported several contexts of parent–child conflict in which CPV occurred. According to the theory of conflict management strategies [19], the contexts of parent–child conflict identified in this study can be categorized as the result of differences in beliefs (e.g., adolescents’ resistance or procrastination in completing tasks), values (e.g., adolescents’ unmet material desires), and priorities (e.g., cell phone usage time) between adolescents and parents. Studies have also demonstrated two domains of the reasons for CPV, including instrumental and reactive reasons [31,35]. CPV for instrumental reasons arises from adolescents’ intention to achieve certain goals, such as resistance to complete tasks, fighting against cell phone use control, and unmet material desires, as identified in this study [31,35]. CPV for reactive reasons refers to adolescents’ reactions to certain approaches to parental discipline, such as the emotionally charged discipline identified in this study [31,35]. Given that each context of conflict and subsequent CPV calls for a different management approach [23], healthcare professionals should understand these contexts for each adolescent with ADHD and their parents, and assist them in developing appropriate communication strategies to prevent the recurrence of CPV.

Contreras et al. posited that the most common forms of CPV include psychological aggression, physical aggression, financial demand, and control/domination [31]. Similar forms of CPV were found in this study among adolescents with ADHD. However, given that this study focused on children’s aggressive behaviors as experienced by parents, financial demand from adolescents with ADHD was not reported. Financial demand refers to behaviors such as stealing parents’ money or forcing parents to pay debts [36]. Parents may reluctantly comply with their child’s financial demands in order to avoid severe conflict, resulting in these demands not being dealt with in a timely manner. Therefore, healthcare professionals should assess various types of CPV so as not to miss the opportunity to intervene if the subtle CPV of financial demand is overlooked.

The present study found that victimized parents are often fearful and overwhelmed due to a lack of mental preparation and effective coping strategies for CPV. Such psychological impacts and a lack of successful coping experience may cause parents to feel helpless and lose their sense of self-esteem, which is not conducive to facing CPV again in the future. Healthcare professionals should assess parents’ risks of being hurt and effectively support families experiencing CPV [26]. Some parents left the scene or used a passive approach to avoid aggravating parent–child conflict, whereas some parents pushed back against their children in an attempt to encourage them to restrain themselves. Although these conflict management styles can have a temporary effect, long-term use can be detrimental to solving fundamental problems in communication between parents and children.

Some parents in this study tried to rationally communicate with their children and analyzed the incidents leading to conflict to help the child understand them. However, if parents only rationalize unilaterally, it may be difficult for an emotionally agitated adolescent to be convinced. Therefore, some parents believed that they should first control their own emotions, encourage their children to express their feelings, and try to put themselves in their children’s shoes to think about the current situation. The parents also advised practicing communicating with their children using real-life examples, even when there is no dispute. In addition to communication with the child, the parents also recommended self-reflection in order to cope with the next disagreement and avoid the recurrence of CPV. Continuously learning parenting skills is essential for parents to help themselves and their children with ADHD. The participants felt that a more authoritative parenting style was conducive to dealing with CPV. Authoritative parenting focuses on building connections and ensuring a warm, encouraging environment [37]; parents who practice this style of parenting set boundaries for their children in a loving way, with realistic expectations and age-appropriate tools and consequences. Conversely, no participants recommended authoritarian parenting for managing CPV. Authoritarian parenting focuses on parents’ authority and is high on control and low on connection, with parents focusing on enforcing rules, punishment, and consequences, rather than on the reasons behind the behavior [37].

Some parents believe that it is essential to seek outside help in a timely manner when dealing with CPV. However, asking for help often makes parents feel incompetent and ashamed, as well as feeling judged by other family members or neighbors [38]. Obedience to parents’ instructions is considered a manifestation of filial piety in traditional Confucianism [39]. Influenced by this concept, traditional Chinese societies believe that to raise children without giving them a good education is the fault of the father. When a child engages in CPV, society perceives it as a result of the parents’ failure to teach the child well. A society influenced by Confucianism also emphasizes interpersonal harmony. When a child is involved in CPV, the general public will deny the value of the family [40]. Further, traditional Chinese culture often asks mothers to take on the responsibility of caring for their children. Mothers may feel overwhelmed when faced with CPV from their children with ADHD. If mothers do not receive appropriate assistance or are blamed, it will seriously undermine their morale and lead to self-doubt.

### 4.1. Implications

Based on the results of this study, we suggest that parents’ experiences of CPV perpetrated by adolescents with ADHD should be assessed routinely. This study found that although CPV experienced by parents of adolescents with ADHD and parents in the general population may differ in frequency and intensity, the basic components are the same, suggesting that effective interventions are likely to work with both types of families. Given that parents’ and adolescents’ perceptions of the family environment and contexts for CPV may differ markedly [41], it is important to understand the process of CPV from both parents’ and adolescents’ perspectives. A qualitative study exploring parents’, adolescents’, and court officers’ perceptions proposed a two-pronged approach for an effective intervention to reduce CPV, which involves (1) addressing specific and theoretically modifiable emotional, behavioral, and psychological factors at the adolescent level; and (2) inducing change in the family system by addressing environmental barriers to seeking treatment and by creating positive family relationships [42]. A review found that parents experiencing CPV have low levels of perceived self-efficacy and difficulties regulating their emotions, display submissive behavior in parent–child interactions, and tend to relativize children’s CPV [43]. Children’s CPV often causes a negative family climate and alters social relations due to a mistrust of formal support resources and a certain level of social isolation [43]. There is a need for interventions aimed at fostering parenting skills and improving formal support resources to enhance family functioning in situations of CPV [43]. Ibabe Erostabe et al. provided a list of 10 online databases of evidence-based intervention programs for CPV from the United States and Europe, reviewing 6 of them [44]. Health professionals such as family physicians, child psychiatrists, and psychologists can help parents follow the structure of these intervention programs. Strengthening parenting skills to improve communication with children and reduce the risk of experiencing CPV is essential. Parents should also learn strategies to help their children express their thoughts and develop problem-solving strategies when dealing with CPV. Healthcare professionals should also discuss ways to seek help with parents experiencing CPV. Finally, others in the community (e.g., religious leaders, teachers, trusted elders) should provide timely assistance to parents who are victims of child violence, e.g., helping parents to calm their children, notifying the Domestic Violence Unit, and providing temporary shelter for parents.

### 4.2. Limitations

This study has several limitations. First, adolescents with ADHD were recruited from outpatient clinics where they were actively receiving pharmacological or psychological therapy. Future studies should investigate whether our findings can be extended to parents of adolescents with ADHD who are not receiving medical treatment. Second, this study excluded the parents of adolescents with ADHD who had comorbid other neurodevelopmental disorders such as intellectual disability and severe autism spectrum disorder. The experiences of parents of adolescents who have comorbid other neurodevelopmental disorders warrant further study. Third, this study did not explore parents’ experience of financial demand by their children. Further study is needed to examine CPV associated with financial demand and its relationships with other forms of CPV against parents of adolescents with ADHD.

## 5. Conclusions

Various contexts of parent–child conflicts in which CPV occurred were identified in this study. Parents reported experiences of psychological aggression, physical aggression, and restrictions on movement. In addition to feelings of distress, fear, and frustration, parents adopted various strategies for coping with adolescents’ CPV, such as leaving the scene, pushing back, rational communication, controlling their own emotions, encouraging their children to express their feelings, and seeking help. The parents suggested that practicing communication with their children using real-life examples and learning parenting skills is essential to the prevention of CPV and avoiding serious consequences. Parents’ experiences with CPV perpetrated by adolescents with ADHD should be routinely investigated and assessed. Several evidence-based intervention programs for CPV have been proposed. Health professionals should help parents to strengthen parenting skills, improve communication with children, and reduce the risk of experiencing CPV.

## Figures and Tables

**Table 1:** Summary of themes and subthemes.

Themes		Subthemes	
1.	The contexts of parent–child conflict in which child-to-parent violence occurred	1.	Resistance or procrastination in completing tasks
		2.	Fighting against cell phone use, control, or daily routines
		3.	Perceived parental favoritism toward siblings
		4.	Unmet material desires
		5.	Unfiltered or emotionally charged discipline
2.	Types of child-to-parent violence	1.	Verbal abuse or threats
		2.	Property destruction
		3.	Physical attacks or throwing objects at parents
		4.	Retaliation against harsh discipline
		5.	Restricting parents’ movement
3.	Parents’ feelings and responses to their children’s violence	1.	Emotional distress, fear, and frustration
		2.	Leaving the scene or using a passive approach
		3.	Using strong reactions to stop the child
		4.	Rational communication
		5.	Thinking from the children’s perspective
		6.	Helping children reduce conflicts with older adults
		7.	Seeking help
4.	Strategies for better management of child-to-parent violence based on past experiences	1.	Controlling their own emotions and leaving the scene
		2.	Avoiding arguments that trigger child violence
		3.	Analyzing situations to help the child understand them
		4.	Encouraging children to express their feelings
		5.	Self-reflection
		6.	Practicing communication in everyday life
		7.	Strengthening parenting skills

## Data Availability

The data are available upon reasonable request to the corresponding authors.
